# Natural DNA Uptake by *Escherichia coli*

**DOI:** 10.1371/journal.pone.0035620

**Published:** 2012-04-19

**Authors:** Sunita Sinha, Rosemary J. Redfield

**Affiliations:** Department of Zoology, University of British Columbia, Vancouver, British Columbia, Canada; University of Massachusetts Medical School, United States of America

## Abstract

*Escherichia coli* has homologues of the competence genes other species use for DNA uptake and processing, but natural competence and transformation have never been detected. Although we previously showed that these genes are induced by the competence regulator Sxy as in other gamma-proteobacteria, no conditions are known that naturally induce *sxy* expression. We have now tested whether the competence gene homologues encode a functional DNA uptake machinery and whether DNA uptake leads to recombination, by investigating the effects of plasmid-borne *sxy* expression on natural competence in a wide variety of *E. coli* strains. High- and low-level *sxy* expression alone did not induce transformation in any of the strains tested, despite varying the transforming DNA, its concentration, and the incubation conditions used. Direct measurements of uptake of radiolabelled DNA were below the limit of detection, however transformants were readily detected when recombination functions were provided by the lambda Red recombinase. This is the first demonstration that *E. coli sxy* expression can induce natural DNA uptake and that *E. coli*'s competence genes do encode a functional uptake machinery. However, the amount of transformation cells undergo is limited both by low levels of DNA uptake and by inefficient DNA processing/recombination.

## Introduction

Many bacteria can actively take up DNA from their environment, a genetically programmed ability called natural competence [1]. When this DNA recombines and changes the cell's genotype, the cell is said to be transformed. Natural competence has never been directly demonstrated in *Escherichia coli*, and most transformation instead relies on artificial permeabilisation to bring DNA into cells [2], [3], [4], [5], [6], [7], [8], [9], [10], [11], [12]. Though this DNA can recombine with the host chromosome using the cell's recombination machinery if RecBCD is disabled, higher levels of recombination can be attained by controlled expression of phage recombination proteins (recombineering) [2], [6], [13], [14], [15]. Several studies have shown modest ‘natural’ uptake of plasmid DNA by *E. coli* under specific conditions, but this appears to bypass the classical DNA uptake machinery (the type IV pilus, TFP) so its relationship to natural competence is unclear [16], [17], [18], [19], [20], [21], [22], [23], [24], [25]. Palchevskiy and Finkel showed that *E. coli* can grow in medium containing DNA as the sole source of carbon and energy and that this growth depends on competence gene homologues, suggesting that *E. coli* can take up DNA, but they could not detect chromosomal transformation [26], [27].

Despite not being naturally transformable, *E. coli* and other *Enterobacteriaceae* have homologues of all but one of the competence genes (*pilF2*) common to their nearest transformable relatives, bacteria in the families *Pasteurellaceae* and *Vibrionaceae* [28], [29]. In the best-studied species, *Haemophilus influenzae* (*Pasteurellaceae*) and *Vibrio cholerae* (*Vibrionaceae*), expression of competence genes is controlled by two positive regulators, CRP and Sxy (also known as TfoX) [30], [31], [32], [33], [34], [35], [36], [37], [38]. The CRP regulator (cAMP receptor protein, also called catabolite activator protein (CAP)) responds to the availability of carbon and energy sources signaled by cAMP; it activates transcription by bending DNA to enhance contacts with RNA polymerase [39], [40], [41]. Much less is known about Sxy's function; it is required for competence-specific transcription of a subset of CRP-dependent promoters (CRP-S promoters) and is thought to make direct contacts with CRP [29].

In *H. influenzae* and *V. cholerae*, Sxy is absolutely required for competence development, and the combination of *sxy* induction and CRP activation are sufficient to induce high levels of competence and transformation [30], [33], [34], [37], [38], [42]. We have previously shown that expression of *E. coli sxy* induces all of its competence-gene homologues, but we were unable to detect natural transformation [29]. This raises the question of whether *E. coli*'s CRP-S regulon is truly a competence regulon (*i.e.* one that encodes machinery used for DNA uptake). In this work, we investigate the ability of *E. coli* Sxy to induce natural competence in a wide variety of strains, using assays of transformation and DNA uptake. We show that although *sxy* expression alone is not sufficient for natural competence, transformation can occur when recombination functions are provided artificially.

## Results

### *sxy* expression in different *E. coli* strains does not induce transformation

We previously showed that expression of plasmid-borne *sxy* in strain BW25113 *sxy::*kan strongly induces all of *E. coli*'s competence gene homologues, but we were unable to detect natural transformation with marked chromosomal DNA [29]. Because extensive strain-to-strain differences in competence and transformability are the norm in other species [43], [44], we investigated the effects of *sxy* expression on transformation in a wide range of *E. coli* strains.

#### K-12 strains

The *sxy* plasmid used in our gene expression study (p*Ecsxy*) [29] was transferred to the K-12 derivatives BW25113, C600 and W3110. IPTG was added to induce *sxy* expression in log phase, and cells were given marked chromosomal DNA before plating on selective and non-selective media. No transformants were ever recovered, despite varying (i) the duration of *sxy* induction/incubation with DNA (10, 30, 60, 90, 120, 180 minutes and overnight), (ii) the DNA concentration (1–20 ug/ml), (iii) the DNA type (chromosomal DNA or PCR product) and/or (iv) the DNA marker (*purK*::tet, *crp*::kan, *fliC*::kan, NalR point mutation). All combinations of assay conditions tested are listed in Table S2.

**Figure 1:**
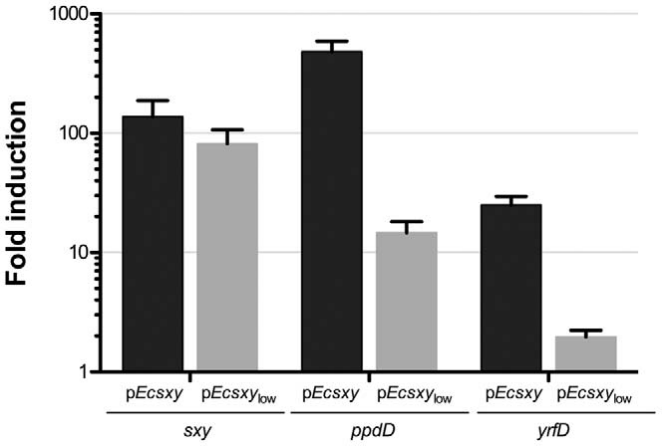
Gene expression upon induction of plasmid-encoded *sxy* genes. qRT-PCR quantification of changes in the expression of *sxy*, *ppdD* and *yrfD* upon *sxy* induction in strain BW25113 after 60 mins expression time. Each bar represents the average of two independent biological replicates for each gene, with error bars indicating standard deviation from the mean. Expression levels shown were normalised for each mRNA sample using 23S rRNA levels.

**Figure 2:**
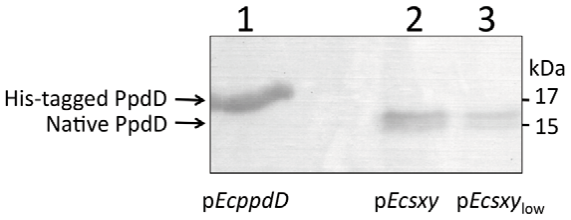
Expression of PpdD pilin protein upon induction of plasmid-encoded *sxy* genes. Western blotting with PpdD antiserum was performed on whole-cell extracts from broth-grown BW25113 60 mins after IPTG induction of *sxy* expression. Lane 1: p*EcppdD* (positive control, His-tagged ppdD); Lane 2: p*Ecsxy*; Lane 3: p*Ecsxy*_low_. The positions of native processed (15 kDa) and His-tagged (17 kDa) PpdD proteins are indicated with arrows.

Sequencing confirmed that the plasmid-encoded *sxy* gene was free of mutations, and qRT-PCR confirmed that *sxy* had been strongly induced upon IPTG addition (Figure 1). The lack of transformation also could not be attributed to failure of Sxy and CRP to induce the CRP-S genes, as we confirmed transcription of *ppdD* and *yrfD* by qRT-PCR (Figure 1, black bars) and production of PpdD pilin by western blotting (Figure 2). Boosting CRP activity by addition of cAMP also had no effect.

We also tested the effect of *sxy* expression in strain ZK126, which Palchevskiy and Finkel showed could use DNA for growth [26], [27]. As in other K-12 strains, *sxy* expression could not mediate natural transformation of ZK126 under any of the conditions described above (Table S2).

#### Other strains

This screen was further extended to natural populations of *E. coli* by investigating the ECOR collection, which is representative of this species' genetic diversity [45], [46]. None of the 72 ECOR strains produced PpdD pilin in overnight or log-phase cultures (data not shown), showing that CRP-S expression (and therefore likely *sxy* expression) is normally low in these strains. Attempts to transform pools of eight strains in overnight culture were also unsuccessful (Table S2). We transferred the *sxy* plasmid to 15 strains chosen to represent diversity in host species, pathogenicity, geographical distribution, subspecific grouping and MSLT type [45], [46]. Again, Sxy expression was never able to mediate chromosomal transformation.

### Moderate *sxy* expression does not induce transformation

Since we had previously observed toxicity with long-term *sxy* expression from the high copy number vector p*Ecsxy*, we reduced expression levels by recloning *E. coli sxy* in the low copy number vector pSU20 to generate p*Ecsxy*_low_. qRT-PCR confirmed *sxy* expression from this plasmid, and showed induction of the CRP-S genes *ppdD* and *yrfD* (Figure 1, grey bars). This expression was lower than from p*Ecsxy*, but still significant. Processed PpdD pilin could be detected by western blotting albeit at reduced levels (Figure 2), suggesting that the competence machinery could still be assembled. All of the transformation conditions described above were retested with p*Ecsxy*_low_, but again *sxy* induction never led to chromosomal transformation.

### Co-expression of *sxy* with *H. influenzae pilF2* does not induce transformation

**Figure 3:**
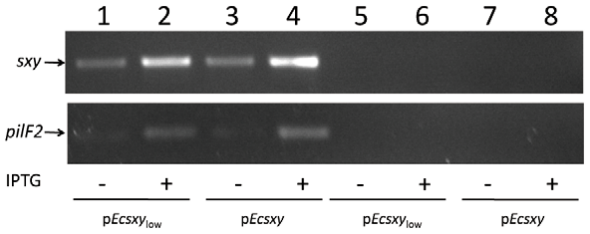
Induction of plasmid-encoded *sxy* and *pilF2* genes in strain ZK126. PCR reactions on cDNA (lanes 1 to 4) confirm that both *sxy* (515 bp; top) and *pilF2* (265 bp; bottom) are strongly expressed when IPTG is added. As a control for DNA contamination of RNA preparations, PCR reactions were also performed on the RNA samples before reverse transcription. As expected, these reactions generated no product (lanes 4 to 8). Lanes 1 and 5: p*Ecsxy*_low_; Lanes 2 and 6: p*Ecsxy*_low_ with IPTG; Lanes 3 and 7: p*Ecsxy*; Lanes 4 and 8: p*Ecsxy* with IPTG.

Although several genes of the *H. influenzae* CRP-S regulon are absent from the *E. coli* genome, only *pilF2* is required for DNA uptake and transformation [28], [29], [47]. This gene is also present in *V. cholerae* and all transformable Pasteurellaceae. Since Pasteurellaceae are the closest relatives of *Enterobacteriaceae*, we cloned the *H. influenzae pilF2* gene downstream of *sxy* in p*Ecsxy* and p*Ecsxy*_low_ and tested whether co-induction of *sxy* and *pilF2* permitted transformation in strain ZK126 under the conditions described above. Despite detectable plasmid-borne expression of both *sxy* and *pilF2* from both plasmids (Figure 3), no transformants were detected (Table S2).

### *E. coli sxy* expression does induce DNA uptake

The inability of diverse *E. coli* strains to be transformed could be due to a lack of DNA uptake across the outer membrane and/or to a defect in DNA processing. This was tested by direct measurement of uptake of radiolabelled chromosomal DNA fragments after *sxy* induction in BW25113 cells. For both p*Ecsxy* or p*Ecsxy*_low_, only 0.02% of the DNA added remained cell-associated after extensive washing (133 cpm per 10^9^ induced cells). This baseline level was higher than background (17 cpm in tubes with no cells), but was not significantly higher than in uninduced cells (107 cpm per 10^9^ cells). When 200 bp DNA fragments were used as donor DNA, levels of cell-associated cpm were not significantly above background (data not shown).

**Figure 4:**
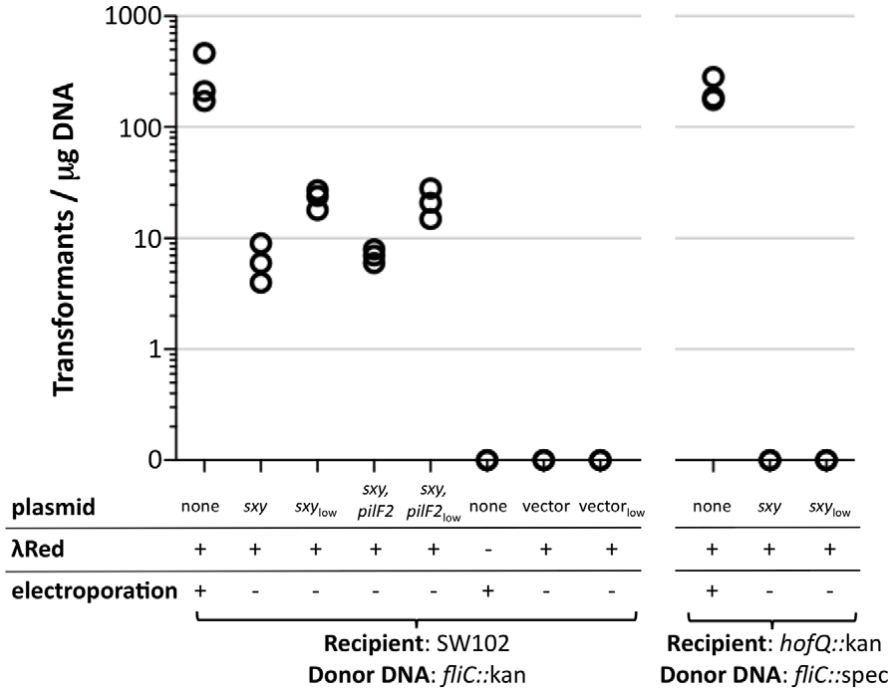
Results of indirect DNA uptake assays. SW102 cells with or without *sxy* plasmids were transformed with 1 µg *fliC*::kan or *fliC*::spec DNA. Cells without plasmids were electroporated. In other cells, *sxy* was induced with IPTG. Lambda Red expression was induced by heat shock. none = no plasmid, *sxy* = high-copy *sxy* plasmid, *sxy*_low_ = low-copy *sxy* plasmid, *sxy,pilF2* = high-copy *sxy-pilF2* plasmid, *sxy,pilF2*_low_ = low-copy *sxy-pilF2* plasmid, vector = high-copy no insert plasmid, vector_low_ = low-copy no insert plasmid.

Since DNA uptake at our detection limit would give substantial numbers of transformants in known naturally transformable bacteria, we developed a more sensitive indirect test of DNA uptake. We measured *sxy*-inducible transformation when recombination functions were provided by the lambda Red recombinase system, which efficiently recombines mutations flanked by short homologous sequences into the chromosome [14]. These experiments used SW102 cells, which carry a chromosomally-encoded Red recombinase [48]. The results of these assays are shown in Figure 4.

In control experiments, SW102 cells were first heat shocked to induce the recombinase and then electroporated with the test DNA fragment, a Kan^R^ cassette flanked by 50 bp segments of chromosomal homology to the *fliC* gene (*fliC::*kan). The transformation efficiency was 284 cfu/µg DNA±160 (Figure 4). To test whether *sxy* expression could induce DNA uptake, we repeated the experiment in SW102 cells carrying p*Ecsxy* or p*Ecsxy*_low_, replacing electroporation with IPTG-induction of *sxy* (Figure 4). Both plasmids gave small but reproducible numbers of Kan^R^ transformants: 6±3 transformants/ug DNA for p*Ecsxy* and 23±5 transformants/ug DNA for p*Ecsxy*_low_. Transformant genotypes were confirmed by PCR. Negative control cells carrying an empty plasmid (no *sxy*), or cells with no heat shock (no recombinase) never gave any transformants.

To confirm that the *sxy*-dependent transformation we observed uses the Sxy-regulated type IV pilus machinery for DNA uptake, we repeated the assay in a strain lacking *hofQ*, which is predicted to encode the outer membrane secretin through which DNA enters competent cells [1], [20], [26], [27], [29], [49], [50], [51]. Because the *hofQ* deletion contains a Kan^R^ cassette, the donor DNA was changed to *fliC::*spec. While SW102 *hofQ::*kan cells electroporated with *fliC::*spec DNA gave 215±60 transformants/ug DNA, cells expressing *sxy* gave none (Figure 4), confirming that the DNA uptake we observed used the competence machinery.

Since transformation following *sxy*-mediated natural DNA uptake was lower than transformation following electroporation, we also tested whether transformation efficiency could be improved by co-expression of *H. influenzae pilF2*, again using plasmids p*Ecsxy-pilF2* and p*Ecsxy-pilF2*_low_. As shown in Figure 4, transformation efficiency was not improved.

These experiments confirm that *E. coli* can indeed take up small amounts of DNA using the DNA uptake machinery, and that this uptake is competence-specific and Sxy-inducible. Collectively, our experiments suggest that natural transformation is undetectable because artificial induction of *sxy* causes very little DNA to be taken up.

## Discussion

*E. coli* can be transformed in the laboratory, but this requires artificial entry of DNA into cells. However *E. coli* should be naturally transformable because it has a set of inducible competence gene homologues in a functional regulon [29]. Here we show that *sxy* expression can lead to natural DNA uptake using the competence machinery. Nevertheless, the amount of DNA uptake is very low, and transformation only occurs when recombination functions are provided artificially, suggesting that recombination of incoming DNA itself is also inefficient. Recombination in *E. coli* may be limited by expression of the RecBCD exonuclease, though studies in *H. influenzae* have shown that transformation is not increased in *recBCD* mutants [6], [52], [53]. The combination of low uptake and limited recombination could explain why researchers have until now been unable to demonstrate natural competence in *E. coli*. A further hurdle is that none of the extensive conditions we and others have tested induce expression of *sxy* or the CRP-S genes it regulates [26], [29], [49], [50].

All competent species studied show extensive strain-to-strain variation in their ability to take up DNA and be transformed [43], [44]. This has caused some species initially classed as non-competent on the basis of one non-transformable isolate to be later re-classified when broader screening identified transformable strains. However none of the *E. coli* strains we tested could undergo chromosomal transformation upon *sxy* expression, so this is not a strain-specific problem.

Since *E. coli*'s CRP-S genes do encode a functional DNA uptake machinery, natural conditions must exist under which higher levels of natural competence occur. Laboratory culture is a very unnatural condition, as is the IPTG induction of *sxy* expression, so it may be that other factors are required which are missing when Sxy is artificially induced. It is therefore important to find more natural conditions that induce *sxy* expression, which requires a better understanding of how it is regulated.

Insights into the regulation of *sxy* expression can be gained from studies in *H. influenzae* and *V. cholerae*, where *sxy* is controlled by both transcriptional and translational regulation [33], [36], [37], [54]. Transcriptional control is mediated by CRP. Like its homologues in *H. influenzae* and *V. cholerae*, *E. coli sxy* has a strong CRP binding site upstream of the transcriptional start site, strongly suggesting that CRP also induces *sxy* transcription in this species. Translational control in *H. influenzae* and *V. cholerae* occurs through the formation of a stem loop structure in *sxy* mRNA that limits the amount of translation [36], [37], [54]. This stem loop is thought to respond to competence-inducing signals, modulating the amount of Sxy protein cells make and thus controlling competence. We have mapped the transcriptional start site of *E. coli sxy* mRNA and found that transcription initiates at a G residue situated 115 bp upstream of the ATG start. Mfold simulations with *E. coli sxy* mRNA predict a stem loop structure very similar to those found in *H. influenzae* and *V. cholerae*. Moreover, Yamamoto et al. identified strong inverted repeats in the promoter region of *V. cholerae sxy*, and showed that these were essential for transcriptional and translational control of *sxy* expression [36], [37]. Such regions of strong base pairing are also found in the region upstream of *H. influenzae* and *E. coli sxy*s, suggesting that similar mechanisms of *sxy* regulation exist in *E. coli* and perhaps all gamma-proteobacteria.

The signals that control *sxy* expression and induce competence in *H. influenzae* and *V. cholerae* are known (purine nucleotide depletion and chitin availability, respectively). Finding the signals that control *sxy* expression in *E. coli* is crucial to finding out how this species becomes competent in its natural environment. This signal is likely to be something that *E. coli* encounters *in vivo*, so it is conceivable that different classes of *E. coli* might respond to slightly different cues. Clearly these conditions are not being met in the laboratory setting.

## Materials and Methods

### Bacterial growth conditions, strains, and plasmids

Details on the strains used in this study are given in Table 1. *E. coli* was grown on Luria Bertani (LB) broth or LB agar (1.2%) at 30°C or 37°C. Plasmids were introduced by CaCl_2_–heat shock transformation. When required, antibiotics were used at the following concentrations: kanamycin 15 µg/ml, chloramphenicol 20 µg/ml, nalidixic acid 20 µg/ml, ampicillin 100 µg/ml, tetracycline 10 µg/ml. Where required, isopropyl β-D-1-thiogalactopyranoside (IPTG) and cyclic adenosine monophosphate (cAMP) were used at 1 mM.

**Table 1:** Bacterial strains used in this study.

Strain name	Genotype	Source/Reference
C600	F- λ- *tonA*21 *thi*-1 *thr*-1 *leuB*6 *lacY*1 *glnV*44 *rfbC*1 *fhuA*1	[11], [59]
W3110	F- λ- *rph*-1 INV(*rrnD*, *rrnE*)	[60]
ZK126	W3110 Δ*lacU*169 *tna*-2	From Steven Finkel, USC Los Angeles, USA
SW102	F- *mcrA* Δ(*mrr-hsdRMS*-*mcrBC*) Φ80d*lacZ*ΔM15 Δ*lacX*74 *deoR recA*1 end*A*1 *araD*139 Δ(*ara*, *leu*)7649 *rpsL nupG* [*λcI*857 (*cro-bio*A) <> *tet*] Δ*galK*	Recombineering strain [48]
NK6051	Hfr Hayes *Δlac-prox*III *thi*-1 *purK*::tn10	From Stan Maloy, University of Illinois, USA
JW5702	BW25113 *crp*::kan	Keio collection [57]
JW1908	BW25113 *fliC*::kan	Keio collection [57]
JW3354	BW25113 *hofQ*::kan	Keio collection [57]
BW25113-NalR	BW25113 NalR	[29]
SW102 *hofQ::*kan	F- *mcrA* Δ(*mrr-hsdRMS*-*mcrBC*) Φ80d*lacZ*ΔM15 Δ*lacX*74 *deoR recA*1 end*A*1 *araD*139 Δ(*ara*, *leu*)7649 *rpsL nupG* [*λcI*857 (*cro-bio*A) <> *tet*] Δ*galK hofQ*::kan	This work
ECOR-10	phylogroup A, subspecies group I, isolated from human in Sweden	ECOR collection [45], [46]
ECOR-15	phylogroup B1, subspecies group I, isolated from human in Sweden	ECOR collection [45], [46]
ECOR-22	phylogroup A, subspecies group I, isolated from animal in Bali	ECOR collection [45], [46]
ECOR-31	phylogroup A, subspecies group II, isolated from animal in USA	ECOR collection [45], [46]
ECOR-34	phylogroup B1, subspecies group II, isolated from animal in USA	ECOR collection [45], [46]
ECOR-35	phylogroup D, subspecies group II, isolated from human in USA	ECOR collection [45], [46]
ECOR-37	phylogroup E, subspecies group II, isolated from animal in USA	ECOR collection [45], [46]
ECOR-42	phylogroup E, subspecies group II, isolated from human in USA	ECOR collection [45], [46]
ECOR-47	phylogroup D, subspecies group II, isolated from animal in New Zealand	ECOR collection [45], [46]
ECOR-48	phylogroup D, subspecies group II, isolated from human in Sweden	ECOR collection [45], [46]
ECOR-55	phylogroup B2, subspecies group III, isolated from human in Sweden	ECOR collection [45], [46]
ECOR-65	phylogroup B2, subspecies group III, isolated from human in USA	ECOR collection [45], [46]
ECOR-67	phylogroup B1, subspecies group III, isolated from animal in Indonesia	ECOR collection [45], [46]
ECOR-72	phylogroup B1, subspecies group III, isolated from human in Sweden	ECOR collection [45], [46]

Strain SW102 *hofQ::*kan was constructed using recombineering to transfer the mutation from the Keio collection strain JW3354 to the chromosome of strain SW102 [48]. Plasmid p*Ecsxy* contains the coding sequence of *E. coli sxy*, cloned downstream of the p-T5-*lac* promoter in the high-copy number vector pCA24N [55]. To create plasmid p*Ecsxy*_low_, an 800 bp segment flanking and including the coding sequence of *E. coli sxy* (b0959) was PCR-amplified from the genome of strain BW25113 using primers with integrated *Cla*I and *Eco*RI restriction sites (all PCR primers are listed in Table S1). The PCR product was digested with *Cla*I and *Eco*RI, and cloned into the low-copy number vector pSU20 digested with the same enzymes, putting *sxy* under control of the *lac* promoter [56].

To create plasmids p*Ecsxy*-*pilF2* and p*Ecsxy*_low_-*pilF2*, the *H. influenzae pilF2* gene (HI0366) was first PCR-amplified and cloned into pGEMT-Easy (Promega) to generate p*HipilF2*. The *pilF2* insert was then released from p*HipilF2* by *Not*I or *Eco*RI digestions, and cloned into the *Not*I site of p*Ecsxy* to generate plasmid p*Ecsxy*-*pilF2*, or the *Eco*RI site of p*Ecsxy*_low_ to generate plasmid p*Ecsxy*_low_-*pilF2*. In both plasmids *pilF2* is cloned in the forward orientation downstream of *E. coli sxy* and under control of the *lac* promoter.

### Transformation assays

Overnight cultures of strains carrying *sxy*-expressing plasmids were diluted 1∶100 in LB-Cm and grown at 37°C. At OD_600_ 0.3, *sxy* expression was induced with IPTG and 1–20 ug/ml donor DNA was added. The donor DNA was either *E. coli* chromosomal DNA or purified PCR product with one of the following genotypes: *purK*::tet, *crp*::kan, *fliC*::kan or BW25113-NalR (see Table 1). Where necessary, cAMP was added at the same time as IPTG. Cells were incubated with DNA for 10, 30, 60, 90, 120 or 180 minutes, or overnight, before being plated on selective and non-selective media. The full list of combinations tested is given in Table S2.

### DNA uptake assays

BW25113 chromosomal DNA was digested with *Xba*I and *Eco*RI and end-labelled with Klenow using ^33^P-dATP. Cultures of strain BW25113 carrying p*Ecsxy* or p*Ecsxylow* were grown at 37°C and *sxy* expression was induced at OD_600_ 0.3 for 30 minutes. Cells were then incubated with 3 µg/ml ^33^P-labelled DNA for 30 minutes at 37°C, pelleted and washed twice with LB, and finally resuspended in LB for scintillation counting.

### Indirect DNA uptake assays

These assays used the recombineering strain SW102, which contains chromosomally encoded *exo*, *bet* and *gam* genes from phage λ, controlled by the inducible *cI*857 promoter [48]. In positive control assays, overnight cultures were diluted in 3 mL LB and grown at 30°C to OD_600_ 0.6, when recombination functions were induced by transfer to 42°C for 15 mins. Cells were then pelleted, washed three times with ice-cold dH_2_0, concentrated 100-fold and electroporated with 1 µg donor DNA. Electroporated cells were allowed to recover in 1 mL LB for 1 h at 30°C before plating.

Assays testing the effect of *sxy* on DNA uptake omitted the electroporation step. Instead, *sxy* expression was induced with IPTG at OD_600_ 0.3 and cells were given 1 µg/ml DNA. When the OD_600_ reached 0.6, recombination functions were induced by heat shock as above. Cells were then returned to 30°C and allowed to grow for a further 1.5 h at 30°C before plating. The donor DNAs used in these experiments were Δ*fliC* PCR products amplified from either the chromosome of the Keio strain JW1908 (*fliC*::kan) (Table 1) [57] or plasmid pRSM2832 (*fliC::*spec) [58].

### Gene and protein expression methods

Expression of plasmid-borne *sxy* and *pilF2* genes was confirmed by RT-PCR (primer sequences are given in Table S1). For each sample, 1 µg RNA was reverse-transcribed with iScript™ (Biorad), and 1 µL cDNA was used in PCR. Quantitative PCR was performed as described in Cameron and Redfield [28], using primers specified in Sinha *et al.* [29] (listed in Table S1). Protein sample preparation and immunoblotting were performed as described in Sinha *et al.* [29].

## Supporting Information

Table S1 Primers used in this study.
(PDF)

Table S2 Conditions tested in transformation assays. None gave any transformants.
(PDF)
