# Supplementary material for: Natural DNA Uptake by Escherichia coli
Source: PLoS One. 2012 Apr 19;7(4):e35620. doi: 10.1371/journal.pone.0035620 (PMC3330819; doi:10.1371/journal.pone.0035620)
Supplement: Table S1 — Primers used in this study. (PDF) [file pone.0035620.s001.pdf]

Primer name	*Description*	*Sequence*
*Ec_sxy*_F	Used to amplify *E. coli sxy* for cloning into p*Ecsxy* (relevant restriction sites are underlined).	CCATCGATTCGTTGGT
*Ec_sxy*_R		CGGAATTCGCATTACTG CCTGAGATTGG
*Hi_pilF2*_F	Used to amplify *H. influenzae pilF2* for cloning into p*Ecsxy* and p*Ecsxy*$_{low}$.	CCAAGTTGATGGGCTTT TTC
*Hi_pilF2*_R		ATTTAGCCTCCATTTACC GC
Δ*fliC*_F	Used to amplify the mutagenic cassette for indirect uptake assays.	GGAAACCCAATACGTAA TCAACGACTTGCAATAT AGGATAACGAATCATGA TTCCGGGGATCCGTCGA CC
Δ*fliC*_R		GTCAGTCTCAGTTAATC AGGTTACAACGATTAAC CCTGCAGCAGAGACAGT GTAGGCTGGAGCTGCTT CG
*sxy*_RT_F	Used to confirm plasmid-encoded *sxy* expression upon IPTG addition.	GCCTCTCCTATAAGCGG ATCTAT
*sxy*_RT_R		GGCACCTTCAAGCATAA ACAGAATCT
*pilF2*_RT_F	Used to confirm plasmid-encoded *pilF2* expression upon IPTG addition.	GGCACGATAAAATGGGC AA
*pilF2*_RT_R		CCATTCCCATCATTACC ACAT
