# Supplementary material for: Natural DNA Uptake by Escherichia coli
Source: PLoS One. 2012 Apr 19;7(4):e35620. doi: 10.1371/journal.pone.0035620 (PMC3330819; doi:10.1371/journal.pone.0035620)
Supplement: Table S2 — Conditions tested in transformation assays. None gave any transformants. (PDF) [file pone.0035620.s002.pdf]

Recipient Strain	Plasmid	Donor DNA genotype	Donor DNA type	Donor DNA concentration (μg/ml)	Time incubated with DNA (minutes)
BW25113	p*Ecsxy*	W3110 Nal^R	chromosomal	1	10, 30, 60, 90, 120, 180, O/N
BW25113	p*Ecsxy*	Hfr hayes *purE::*tet	chromosomal	1	30, 60, 90, 120, 180, O/N
BW25113	p*Ecsxy*	BW25113 *crp::*kan	chromosomal	1	30, 60, 90, 120, 180, O/N
BW25113	p*Ecsxy*	W3110 Nal^R	chromosomal	10	30, 60, 90, 120, 180, O/N
BW25113	p*Ecsxy*	Hfr hayes *purE::*tet	chromosomal	10	30, 60, 90, 120, 180, O/N
BW25113	p*Ecsxy*	BW25113 *crp::*kan	chromosomal	10	30, 60, 90, 120, 180, O/N
BW25113	p*Ecsxy*	W3110 Nal^R	chromosomal	20	60, 120, 180
BW25113	p*Ecsxy*	Hfr hayes *purE::*tet	chromosomal	20	60, 120, 180
BW25113	p*Ecsxy*	BW25113 *crp::*kan	chromosomal	20	60, 120, 180
BW25113	p$Ecsxy_{low}$	W3110 Nal^R	chromosomal	1	30, 60, 90, 120, 180, O/N
BW25113	p$Ecsxy_{low}$	Hfr hayes *purE::*tet	chromosomal	1	30, 60, 90, 120, 180, O/N
BW25113	p$Ecsxy_{low}$	BW25113 *crp::*kan	chromosomal	1	30, 60, 90, 120, 180, O/N
BW25113	p$Ecsxy_{low}$	W3110 Nal^R	chromosomal	10	60, 120, 180, O/N
BW25113	p$Ecsxy_{low}$	Hfr hayes *purE::*tet	chromosomal	10	60, 120, 180, O/N
BW25113	p$Ecsxy_{low}$	BW25113 *crp::*kan	chromosomal	10	60, 120, 180, O/N
BW25113	p$Ecsxy_{low}$	W3110 Nal^R	chromosomal	20	60, 120, 180, O/N
BW25113	p$Ecsxy_{low}$	Hfr hayes *purE::*tet	chromosomal	20	60, 120, 180, O/N
BW25113	p$Ecsxy_{low}$	BW25113 *crp::*kan	chromosomal	20	60, 120, 180, O/N

BW25113	p*Ecsxy*	BW25113 *crp::*kan	PCR	10	60, 120, O/N
BW25113	p*Ecsxy*low	BW25113 *crp::*kan	PCR	10	60, 120, O/N
W3100	p*Ecsxy*	BW25113 *crp::*kan	chromosomal	15	60, 120, 180, O/N
W3110	p*Ecsxy*	BW25113 *crp::*kan	PCR	10	60, 120, 180, O/N
W3110	p*Ecsxy*	BW25113 *crp::*kan	PCR	4	120
W3110	p*Ecsxy*	BW25113 *fliC::*kan	PCR	4	120
W3110	p*Ecsxy*	BW25113 *fliC::*kan	chromosomal	10	120
W3110	p*Ecsxy*	W3110 Nal^R	chromosomal	10	120
W3100	p*Ecsxy*low	BW25113 *crp::*kan	chromosomal	15	120, O/N
W3110	p*Ecsxy*low	BW25113 *crp::*kan	PCR	10	120, O/N
W3110	p*Ecsxy*low	BW25113 *crp::*kan	PCR	4	120
W3110	p*Ecsxy*low	BW25113 *fliC::*kan	PCR	4	120
W3110	p*Ecsxy*low	BW25113 *fliC::*kan	chromosomal	10	120
W3110	p*Ecsxy*low	W3110 Nal^R	chromosomal	10	120
C600	p*Ecsxy*	BW25113 *crp::*kan	chromosomal	15	60, 120, 180, O/N
C600	p*Ecsxy*	BW25113 *crp::*kan	PCR	10	60, 120, 180, O/N
C600	p*Ecsxy*	BW25113 *fliC::*kan	PCR	4	120
C600	p*Ecsxy*	BW25113 *fliC::*kan	chromosomal	10	120
C600	p*Ecsxy*	W3110 Nal^R	chromosomal	10	120
C600	p*Ecsxy*low	BW25113 *crp::*kan	chromosomal	15	120, O/N
C600	p*Ecsxy*low	BW25113 *crp::*kan	PCR	10	60, 120, 180, O/N
C600	p*Ecsxy*low	BW25113 *fliC::*kan	chromosomal	10	120
C600	p*Ecsxy*low	W3110 Nal^R	chromosomal	10	120
C600	p*Ecsxy*low	W3110 Nal^R	chromosomal	10	120
ZK126	p*Ecsxy*	BW25113	chromosomal	1	120, O/N

		*crp::*kan			
ZK126	p*Ecsxy*	BW25113 *crp::*kan	chromosomal	10	120, O/N
ZK126	p*Ecsxy*	BW25113 *crp::*kan	PCR	10	120, O/N
ZK126	p*Ecsxy*	BW25113 *crp::*kan	PCR	20	180
ZK126	p*Ecsxy*	BW25113 *fliC::*kan	chromosomal	10	120, O/N
ZK126	p*Ecsxy*	W3110 Nal^R	chromosomal	10	120, O/N
ZK126	p*Ecsxy*$_{low}$	BW25113 *crp::*kan	chromosomal	1	120, O/N
ZK126	p*Ecsxy*$_{low}$	BW25113 *crp::*kan	chromosomal	10	120, O/N
ZK126	p*Ecsxy*$_{low}$	BW25113 *crp::*kan	PCR	10	120, O/N
ZK126	p*Ecsxy*	BW25113 *crp::*kan	PCR	20	180
ZK126	p*Ecsxy*$_{low}$	BW25113 *fliC::*kan	chromosomal	10	120, O/N
ZK126	p*Ecsxy*$_{low}$	W3110 Nal^R	chromosomal	10	120, O/N
ZK126	p*Ecsxy-pilF2*	BW25113 *crp::*kan	chromosomal	10	60, 120, O/N
ZK126	p*Ecsxy-pilF2*	BW25113 *crp::*kan	PCR	10	60, 120, O/N
ZK126	p*Ecsxy-pilF2*	BW25113 *crp::*kan	chromosomal	10	120, O/N
ZK126	p*Ecsxy*$_{low}$*-pilF2*	BW25113 *crp::*kan	chromosomal	10	60, 120, O/N
ZK126	p*Ecsxy*$_{low}$*-pilF2*	BW25113 *crp::*kan	PCR	10	60, 120, O/N
ZK126	p*Ecsxy*$_{low}$*-pilF2*	BW25113 *crp::*kan	chromosomal	10	120, O/N
ECOR01	none	BW25113 *crp::*kan	chromosomal	1	O/N
ECOR01	none	BW25113 *crp::*kan	chromosomal	10	O/N
ECOR02	none	BW25113 *crp::*kan	chromosomal	10	O/N
ECOR03	none	BW25113 *crp::*kan	chromosomal	10	O/N
ECOR04	none	BW25113 *crp::*kan	chromosomal	10	O/N
ECOR05	none	BW25113 *crp::*kan	chromosomal	10	O/N
ECOR23, 24, 26, 27,	none	BW25113 *crp::*kan	chromosomal	10	O/N

28, 30, 34, 39					
ECOR11, 21, 31, 41, 51, 61, 71	none	BW25113 *crp::*kan	chromosomal	10	O/N
ECOR31	p*Ecsxy*	BW25113 *crp::*kan	chromosomal	10	180
ECOR22	p*Ecsxy*	BW25113 *crp::*kan	chromosomal	10	180
ECOR34	p*Ecsxy*	BW25113 *crp::*kan	chromosomal	10	180
ECOR72	p*Ecsxy*	BW25113 *crp::*kan	chromosomal	10	180
ECOR55	p*Ecsxy*	BW25113 *crp::*kan	chromosomal	10	180
ECOR65	p*Ecsxy*	BW25113 *crp::*kan	chromosomal	10	180
ECOR38	p*Ecsxy*	BW25113 *crp::*kan	chromosomal	10	180
ECOR39	p*Ecsxy*	BW25113 *crp::*kan	chromosomal	10	180
ECOR40	p*Ecsxy*	BW25113 *crp::*kan	chromosomal	10	180
ECOR41	p*Ecsxy*	BW25113 *crp::*kan	chromosomal	10	180
ECOR43	p*Ecsxy*	BW25113 *crp::*kan	chromosomal	10	180
ECOR35	p*Ecsxy*	BW25113 *crp::*kan	chromosomal	10	180
ECOR36	p*Ecsxy*	BW25113 *crp::*kan	chromosomal	10	180
ECOR37	p*Ecsxy*	BW25113 *crp::*kan	chromosomal	10	180
ECOR42	p*Ecsxy*	BW25113 *crp::*kan	chromosomal	10	180
ECOR31	p$Ecsxy_{low}$	BW25113 *crp::*kan	chromosomal	10	180
ECOR22	p$Ecsxy_{low}$	BW25113 *crp::*kan	chromosomal	10	180
ECOR34	p$Ecsxy_{low}$	BW25113 *crp::*kan	chromosomal	10	180
ECOR72	p$Ecsxy_{low}$	BW25113 *crp::*kan	chromosomal	10	180
ECOR55	p$Ecsxy_{low}$	BW25113 *crp::*kan	chromosomal	10	180
ECOR65	p$Ecsxy_{low}$	BW25113 *crp::*kan	chromosomal	10	180
ECOR38	p$Ecsxy_{low}$	BW25113 *crp::*kan	chromosomal	10	180

ECOR39	p*Ecsxy*$_{low}$	BW25113 *crp::*kan	chromosomal	10	180
ECOR40	p*Ecsxy*$_{low}$	BW25113 *crp::*kan	chromosomal	10	180
ECOR41	p*Ecsxy*$_{low}$	BW25113 *crp::*kan	chromosomal	10	180
ECOR43	p*Ecsxy*$_{low}$	BW25113 *crp::*kan	chromosomal	10	180
ECOR35	p*Ecsxy*$_{low}$	BW25113 *crp::*kan	chromosomal	10	180
ECOR36	p*Ecsxy*$_{low}$	BW25113 *crp::*kan	chromosomal	10	180
ECOR37	p*Ecsxy*$_{low}$	BW25113 *crp::*kan	chromosomal	10	180
ECOR42	p*Ecsxy*$_{low}$	BW25113 *crp::*kan	chromosomal	10	180
